# Neuroprotective Effects of A Standardized Flavonoid Extract of Safflower Against Neurotoxin-Induced Cellular and Animal Models of Parkinson’s Disease

**DOI:** 10.1038/srep22135

**Published:** 2016-02-24

**Authors:** Rutong Ren, Chunyan Shi, Jing Cao, Yi Sun, Xin Zhao, Yongfei Guo, Chen Wang, Hui Lei, Hanjie Jiang, Nuramatjan Ablat, Jiamin Xu, Wan Li, Yingcong Ma, Xianrong Qi, Min Ye, Xiaoping Pu, Hongbin Han

**Affiliations:** 1 Department of Molecular and Cellular Pharmacology, School of Pharmaceutical Sciences, Peking University, Beijing 100191, China; 2 State Key Laboratory of Natural and Biomimetic Drugs, Peking University, Beijing 100191, China; 3 Beijing Key Lab of MRI Device and Technique, Beijing 100191, China; 4 Department of Radiology, Peking University Third Hospital, Beijing 100191, China

## Abstract

Safflower has long been used to treat cerebrovascular diseases in China. We previously reported that kaempferol derivatives of safflower can bind DJ-1, a protein associated with Parkinson’s disease (PD), and flavonoid extract of safflower exhibited neuroprotective effects in a 1-methyl-4-phenyl-1,2,3,6-tetrahydropyridine-induced mouse model of PD. In this study, a standardized safflower flavonoid extract (SAFE) was isolated from safflower and mainly contained flavonoids. Two marker compounds of SAFE, kaempferol 3-O-rutinoside and anhydrosafflor yellow B, were proven to suppress microtubule destabilization and decreased cell area, respectively. We confirmed that SAFE in dripping pill form could improve behavioural performances in a 6-hydroxydopamine (6-OHDA)-induced rat model of PD, partially via the suppression of α-synuclein overexpression or aggregation, as well as the suppression of reactive astrogliosis. Using an MRI tracer-based method, we found that 6-OHDA could change extracellular space (ECS) diffusion parameters, including a decrease in tortuosity and the rate constant of clearance and an increase in the elimination half-life of the tracer in the 6-OHDA-lesioned substantia nigra. SAFE treatment could partially inhibit the changes in ECS diffusion parameters, which might provide some information about neuronal loss and astrocyte activation. Consequently, our results indicate that SAFE is a potential therapeutic herbal product for treatment of PD.

Parkinson’s disease (PD) is the second most common disorder of the central nervous system (CNS), and its incidence is increasing among people over the age of 60 years^1^. PD is pathologically characterized by the loss of dopaminergic neurons in the substantia nigra (SN) and the formation of cytoplasmic inclusion bodies; however, the aetiology of PD remains elusive. The clinical features of PD include muscular rigidity, resting tremor, bradykinesia, and postural instability. By the time patients are diagnosed with PD, approximately 80% of the striatal dopamine terminals have been lost^2^, and destruction of terminal fields may precede cell body loss in the SN^3^. In rats, the unilateral intracerebral injection of 6-hydroxydopamine (6-OHDA) results in a selective degeneration of dopaminergic neurons, and this is a widely used animal model of PD. 6-OHDA induces a neurodegenerative process in the nigrostriatal system through the inhibition of mitochondrial complex function, which can lead to the induction of oxidative stress, inflammation^4^,^5^,^6^, abnormal protein aggregation^7^,^8^, elevated iron levels^9^ and ultimately cell death.

Dopamine replacement therapy remains the first line strategy in PD treatment. However, its effectiveness cannot modify the progression of the neurodegenerative process. Additionally, dopamine replacement therapy is associated with side-effects that include fluctuations in motor response and dyskinesia^10^. Increasing attention has been placed on Chinese herbal neuroprotective treatments because these treatments are associated with fewer adverse reactions and have exhibited efficacy in the treatment of PD^11^. For example, the aqueous extract from *Decalepis hamiltonii* attenuated neuromotor deficits and suppressed the formation of reactive oxygen species in an A30P and A53T α-synuclein (α-syn) transgenic *Drosophila* model of PD^12^. *Carthamus tinctorius* L. (safflower) is a traditional Chinese medicine (TCM) widely used for the treatment of cardiovascular, cerebrovascular and gynaecological diseases. More than 200 compounds have been extracted and separated from safflower including flavonoids, chalcones, phenylethanoid glycosides, coumarins, steroids and safflower polysaccharides^13^. Phytochemical and pharmacological research has shown that flavonoids exhibit therapeutic effects, including anti-oxidant^14^, anti-coagulant^14^, anti-inflammatory^15^,^16^, and neuroprotective effects^15^,^17^. In previous studies, our group has confirmed that the compounds isolated from safflower, kaempferol 3-O-rutinoside (K3R, Fig. 1a) and anhydrosafflor yellow B (AYB, Fig. 1b), can reduce the levels of hydrogen peroxide (H_2_O_2_)-induced reactive oxygen species and restore tyrosine hydroxylase (TH) activity in PC12 cells^14^. Furthermore, we have also found that flavonoid extracts of safflower exert a neuroprotective effect in a 1-methyl-4-phenyl-1,2,3,6-tetrahydropyridine (MPTP)-induced mouse model of PD^18^,^19^.

Brain tissue essentially has two components: cellular elements (neurons and glial cells), and the gaps between the cellular elements, which are known as the extracellular space (ECS) and resemble the water phase of a foam^20^. The width of the ECS is approximately 20–60 nm^21^. In total, the ECS occupies approximately 20% of the entire tissue volume^20^. The ECS includes ions, transmitters, metabolites, peptides, neurohormones, and other neuroactive substances. Molecules in the ECS directly or indirectly affect neuronal and glial cell function^20^,^22^. Neurons and glial cells release a number of neuroactive substances into the ECS, and then these substances diffuse through the ECS to their targets, which are located on neurons and glial cells and are frequently distant from the release sites. This type of extrasynaptic transmission (i.e., transmission mediated by the diffusion of neuroactive substances through the ECS) is also called “diffusion transmission”^23^ and provides a mechanism for long-term information processing involved in functions such as vigilance, sleep, depression, memory formation, and CNS plasticity^24^. The aforementioned evidence makes diffusion transmission an important area for neuroscience research. Diffusion experiments quantify four ECS parameters (Supplementary Table S1)^20^ that can be altered by cell proliferation, glial cell maturation, cell swelling and neuronal loss^20^. The common physiological processes of PD include dopaminergic neuron loss, protein deposition, and astrocyte activation. These processes might also be related to changes in the ECS. Therefore, determining changes in the ECS diffusion parameters might help provide invaluable insight into the mechanisms and pathologic changes that underlie PD, and further reveal therapeutics for PD treatment.

Thus, the present study was designed to investigate the neuroprotective effect of a standardized flavonoid extract of safflower (SAFE) using a PD cell model that is induced by rotenone and a PD rat model that is induced by 6-OHDA. Additionally, a novel magnetic resonance imaging (MRI) tracer-based method was applied to determine ECS diffusion parameters, which assessed the neuroprotective effects of SAFE in a 6-OHDA-induced rat model of PD.

## Results

### Effects of K3R and AYB on the cell viability of rotenone-treated differentiated PC12 cells

**Figure 1:**
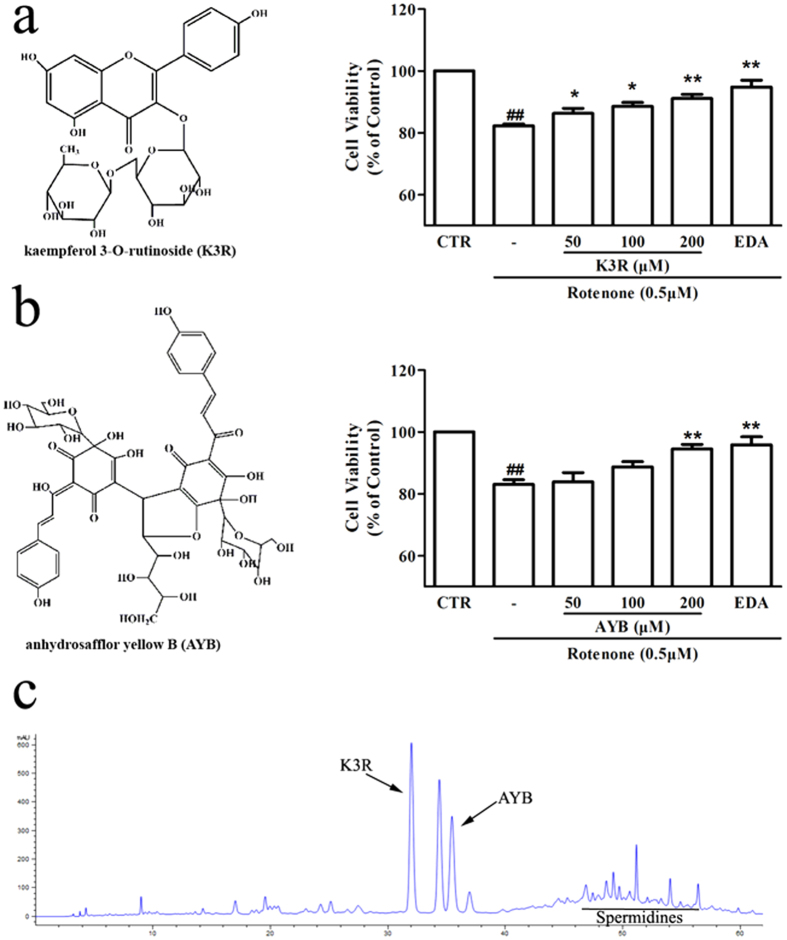
(**a**) Effects of K3R on cell viability of rotenone-induced differentiated PC12 cell damage. (**b**) Effects of AYB on cell viability of rotenone-induced differentiated PC12 cell damage. (**c**) Standardized HPLC chromatogram of SAFE. Cells were pretreated with K3R or AYB (50, 100 and 200 μM) for 6 h before rotenone treatment for 24 h. Data are the mean ± SEM, n = 4–5, ^##^*P* < 0.01 vs. untreated control (CTR) group; *P < 0.05, **P < 0.01 vs. rotenone group.

Prior to evaluating the effects of K3R and AYB on rotenone-induced cell damage, differentiated PC12 cells were pretreated with K3R or AYB at concentrations ranging from 50 to 200 μM for 6 h and subsequently treated with 0.5 μM rotenone for 24 h. As shown in Fig. 1a, within 24 h of treatment with rotenone alone, cell viability was significantly decreased (*P* < 0.01). However, the viability of PC12 cells that were pretreated with K3R significantly increased at K3R concentrations of 50, 100 and 200 μM compared with the cells that were subjected to rotenone treatment alone (*P* < 0.05, *P* < 0.05 and *P* < 0.01, respectively, Fig. 1a). When the samples were pretreated with various concentrations of AYB for 6 h, cell viability markedly increased at the concentration of 200 μM AYB (*P* < 0.01, Fig. 1b).

### High content analysis (HCA) of the protective effects of K3R and AYB against rotenone-induced differentiated PC12 cell damage

**Figure 2:**
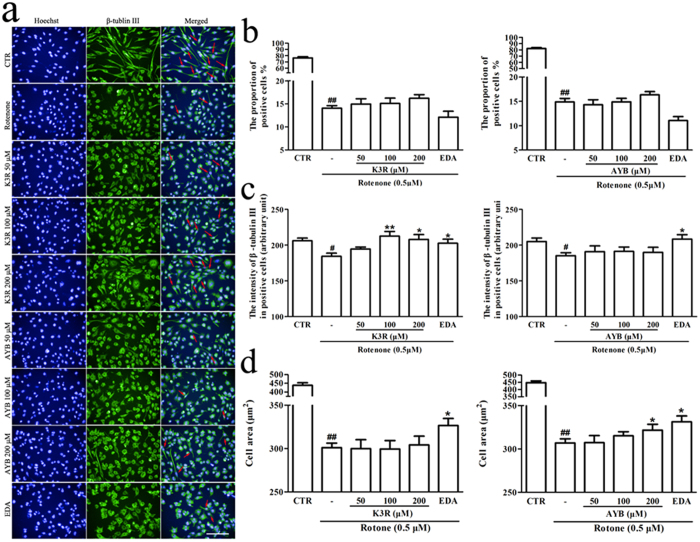
(**a**) High-content images of fluorescently-labeled β-tubulin III in differentiated PC12 cells pretreated with K3R or AYB after rotenone-induced damage. Microtubule structure (green) was visualized by fluorescent labeling using anti-β-tubulin III. Nuclei were counterstained using Hoescht33342 (blue). (**b**) Effects of K3R and AYB on the proportion of positive cells after rotenone-induced damage. (**c**) Effects of K3R and AYB on the fluorescent intensity for β-tubulin III in the positive cells after rotenone-induced damage. (**d**) Effects of K3R and AYB on the cell area after rotenone-induced damage. HCA was analyzed with the Columbus™ software. The cells that were greater than 38 μm in length and had neurite were defined as the positive cells (solid arrows). Cells were pretreated with K3R or AYB (50, 100 and 200 μM) for 6 h before rotenone treatment for 24 h. Bar = 100 μm. Data are the mean ± SEM, n = 9, ^##^*P* < 0.01 vs. untreated control (CTR) group; *P < 0.05, **P < 0.01 vs. rotenone group.

Hoechst staining provides information about cell counts, and β-tubulin III (a marker of cell bodies and all neurites) effectively stains neurites and enables quantitative analysis of important parameters. As shown in Fig. 2a, treatment with rotenone alone caused morphological changes, including shrinkage of the cell bodies and disappearance of the neurites in differentiated PC12 cells. Moreover, the proportion of positive cells (solid arrows, Fig. 2a), the fluorescence intensity for β-tubulin III in the positive cells, and the cell area were significantly decreased (*P* < 0.01, Fig. 2b; *P* < 0.05, Fig. 2c; *P* < 0.01, Fig. 2d). Two marker compounds, K3R and AYB, have no effect on the positive cell level. However, K3R markedly increased the fluorescence intensity of β-tubulin III in the positive cells at concentrations of 100 and 200 μM (*P* < 0.01 and *P* < 0.05, respectively, Fig. 2c) and AYB significantly suppressed the decreased cell area induced by rotenone (*P* < 0.05, Fig. 2d).

### Qualitative and quantitative analyses of SAFE components and preparation of the dripping pills

Figure 1c shows the standardized high performance liquid chromatography (HPLC) chromatogram of SAFE. Our results showed that the main components were flavonoids, which accounted for 62.1% of SAFE. K3R and AYB were representative compounds of flavonoid glycosides and quinochalcones (two flavonoid subclasses), respectively. From the HPLC and liquid chromatography/mass spectrometry (LC/MS) analyses, the extract was found to be composed of approximately 5.6% K3R, 5.7% AYB, and other less prevalent flavonoids, including kaempferol 3-O-β-D-sophoroside (K3S), 6-hydroxykaempferol 3-O-β-D-glucoside (6HK3G), quercetin 3-O-rutinoside (rutin), qucertin-3-O-glucoside (Q3G), 6-hydroxyapigenin 6-O-β-D-glucoside-7-O-β-D-glucuronide (6H6G7G), 4′,5-dihydroxyl-6-O-glucopyranosyl flavanone (4D6GF), kaempferol 3-O-glucoside (K3G) and isorhamnetin (Supplementary Fig. S1 and Table S2). We inferred that the underlined peak (Fig. 1c) might be seldom reported spermidines^25^.

Additionally, the SAFE dripping pills were uniform in shape (round) and colour and exhibited no adhesion phenomena. The average weight of the pills was 42.6 ± 2.6 mg (n = 100) with a relative standard deviation (RSD) of 6.07%. The average disintegration time of the pills in water was 10.8 ± 2 min (n = 6). The appearances, weights, and disintegration times all met the standards of the Chinese Pharmacopoeia (CP)^26^.

### Effects of SAFE on apomorphine-induced rotational behavior in the 6-OHDA-induced rat model of PD

**Figure 3:**
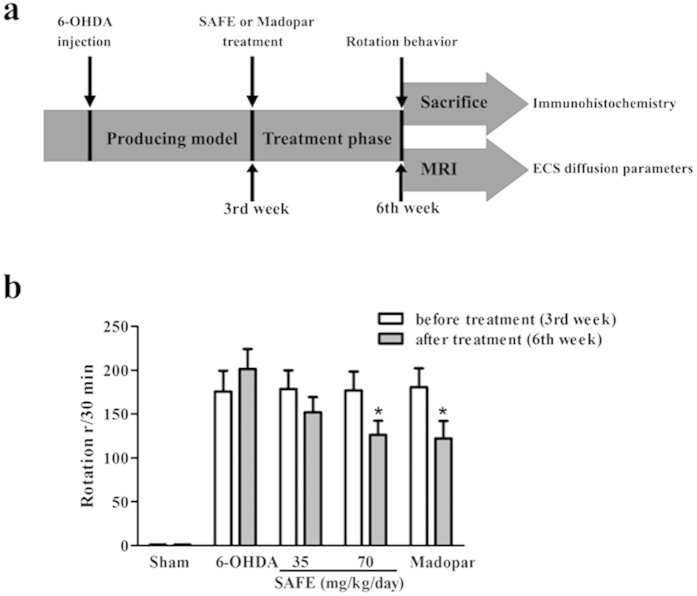
(**a**) The flow chart of rat experiment. (**b**) Effects of SAFE on apomorphine-induced rotational behaviour in the 6-OHDA-induced rat model of PD. Measurements of rotational behavior were conducted before treatment and three weeks after SAFE treatment. Data are the mean ± SEM, n = 10. Statistical analyses were performed using one-way ANOVA. *P < 0.05 vs. 6-OHDA group.

The effects of SAFE were first evaluated in the 6-OHDA-induced rat model of PD, which is a classic PD animal model. After three weeks of SAFE treatment, rats in each group were assessed for rotational behaviour (Fig. 3a). Apomorphine injections caused marked turning in the 6-OHDA group rats (Fig. 3b). Rotations in the SAFE (70 mg/kg/day) and Madopar groups were significantly decreased (both *P* < 0.05, Fig. 3b) compared with the 6-OHDA group of rats.

### Effects of SAFE on TH-positive dopaminergic neurons in the SN of 6-OHDA-induced rats

**Figure 4:**
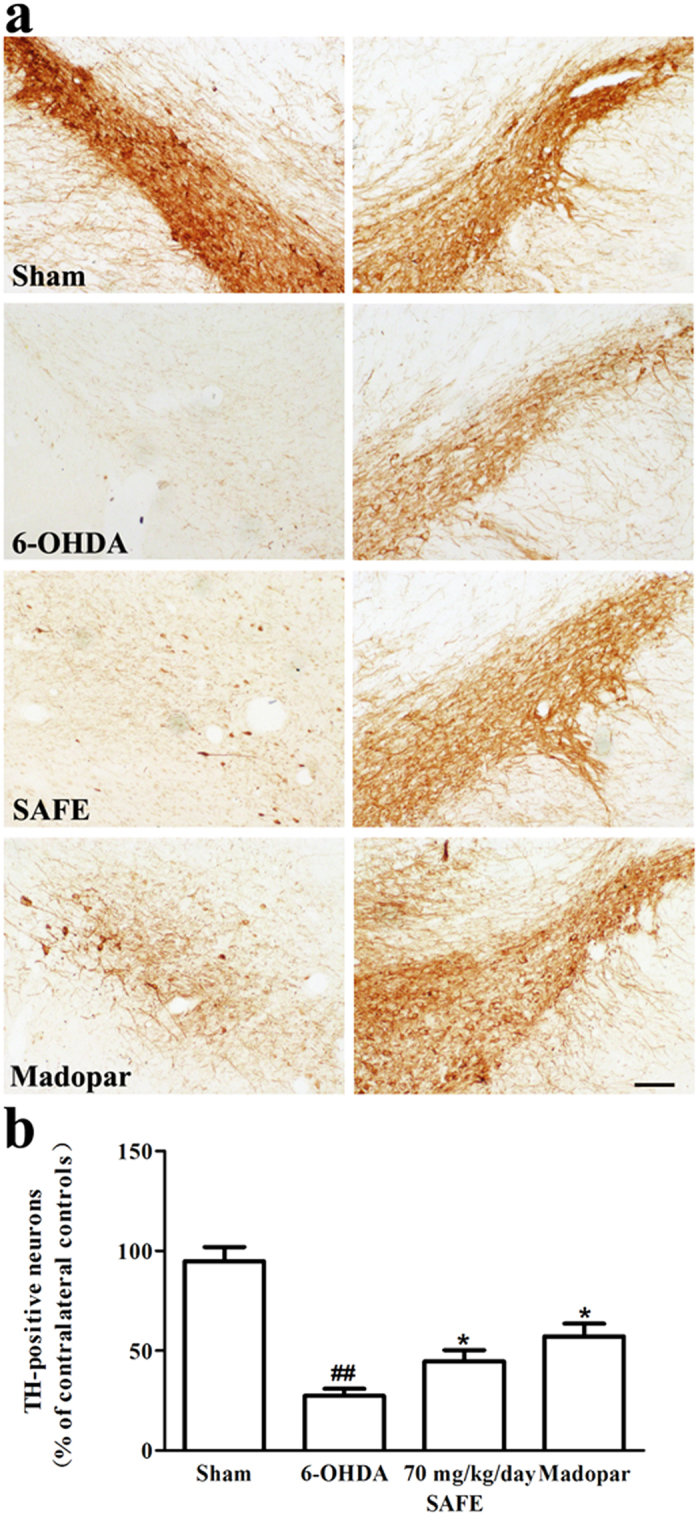
Effects of SAFE on TH-positive dopaminergic neurons in the SN of 6-OHDA-induced rat model of PD. (**a**) Representative photographs showing the appearance of TH-positive neurons. (**b**) Quantitative analysis of TH-positive neurons. Bar = 100 μm. Data are the mean (% of contralateral controls) ± SEM, n = 3–4, and 4 sections per rat. Statistical analyses were performed using one-way ANOVA. ^##^*P* < 0.01 vs. sham group; *P < 0.05 vs. 6-OHDA group.

TH staining was performed to evaluate dopaminergic neuron survival. Representative microphotographs of TH immunostaining in the SN are shown in Fig. 4a. TH-immunoreactive neurons were easily detectable in the SN of the sham group, and the cytoplasm and fibres of the dopaminergic neurons were intensely stained. In contrast, the ratio of TH-immunoreactive neurons in the lesioned side *versus* the intact side were significantly decreased in rats that had been subjected to 6-OHDA injection compared with rats that did not receive 6-OHDA injection (*P* < 0.01, Fig. 4b); conversely, the loss of TH-immunopositive neurons in the lesioned side was significantly inhibited in the rats that received SAFE or Madopar treatment (both *P* < 0.05, Fig. 4b). There also existed more remaining TH-positive fibre in the SAFE and Madopar groups compared with the 6-OHDA group. Thus, it was suggested that SAFE could protect the dopaminergic neurons from 6-OHDA neurotoxicity.

### Effects of SAFE on α-syn in the SN of 6-OHDA-induced rats

**Figure 5:**
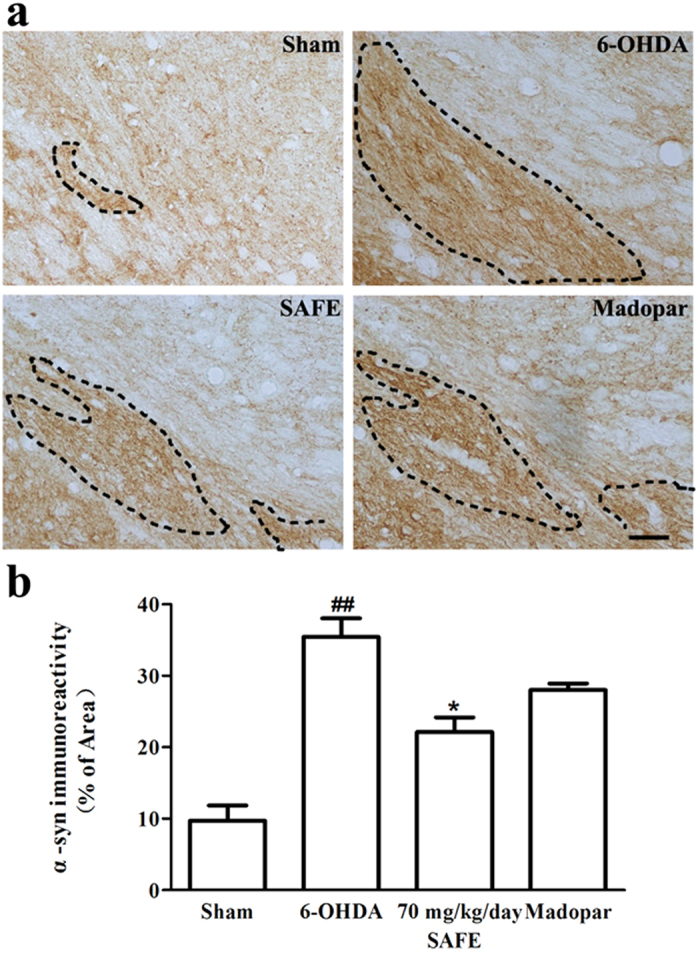
Effects of SAFE on α-syn in the SN of 6-OHDA-induced rat model of PD. (**a**) Representative photographs showing the appearance of α-syn-positive stain. (**b**) Semi-quantitative analysis of α-syn-positive stain. Bar = 50 μm. Data are the mean (% of area) ± SEM, n = 3–4, and 4 sections per rat. Statistical analyses were performed using one-way ANOVA. ^##^*P* < 0.05 vs. Sham group; *P < 0.05 vs. 6-OHDA group.

α-syn aggregation is a neuropathological hallmark of PD and other synucleinopathies. To determine the effects of SAFE on α-syn overexpression or aggregation induced by 6-OHDA, immunohistochemical analyses of α-syn were performed. In the sham group, relatively faint α-syn immunoreactivity was identifiable (Fig. 5a). The area of dark-staining α-syn immunoreactivity (dashed line, Fig. 5a) markedly increased in the 6-OHDA-lesioned SN (*P* < 0.01, Fig. 5b), which indicated that 6-OHDA could induce the overexpression or aggregation of α-syn. Treatment with 70 mg/kg/day SAFE effectively inhibited the 6-OHDA-induced overexpression or aggregation of α-syn (*P* < 0.05, Fig. 5b), but Madopar administration had no significant effect on the overexpression or aggregation of α-syn induced by 6-OHDA.

### Effects of SAFE on astrocytes in the SN of 6-OHDA-induced rats

**Figure 6:**
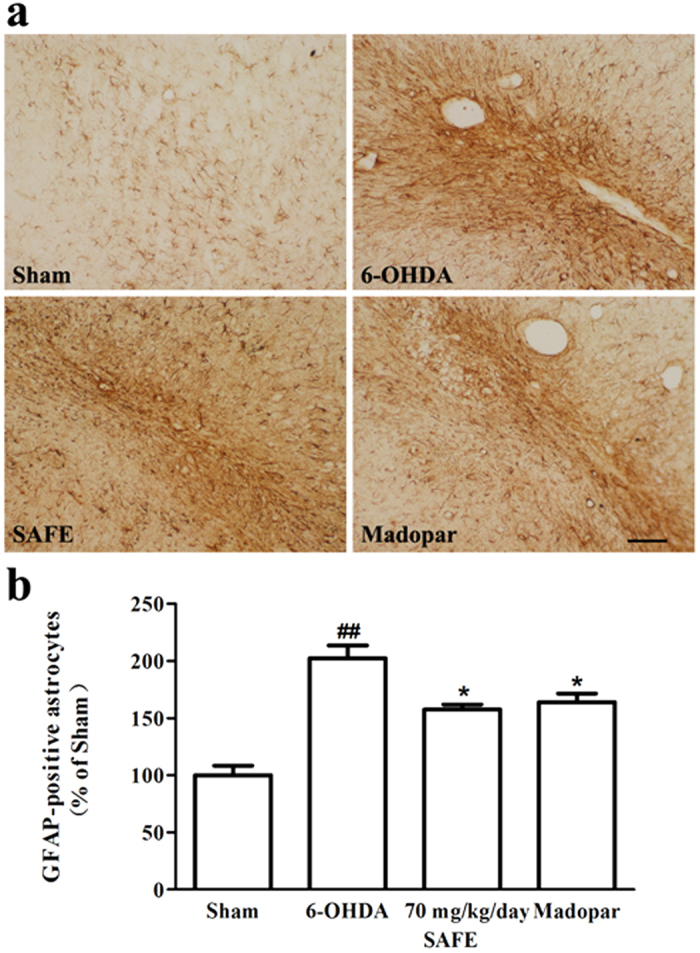
Effects of SAFE on astrocytes in the SN of 6-OHDA-induced rat model of PD. (**a**) Representative photographs showing the appearance of GFAP-positive astrocytes. (**b**) Quantitative analysis of GFAP-positive astrocytes. Bar = 100 μm. Data are the mean (% of sham) ± SD, n = 3–4, and 4 sections per rat. Statistical analyses were performed using one-way ANOVA. ^##^*P* < 0.01 vs. Sham group; *P < 0.05 vs. 6-OHDA group.

The increased expression of glial markers, particularly glial fibrillary acidic protein (GFAP), is a hallmark of reactive astrocytes in the CNS. Fig. 6a shows that immunostaining for GFAP resulted in brown-coloured staining, which represented reactive GFAP-positive astrocytes. The numbers of GFAP-positive astrocytes markedly increased in the 6-OHDA-lesioned SN (*P* < 0.01, Fig. 6b) and the increase was significantly diminished by 70 mg/kg/day SAFE or Madopar treatment (both *P* < 0.05, Fig. 6b).

### Effects of SAFE on ECS diffusion parameters in the SN of 6-OHDA-induced rats

**Figure 7:**
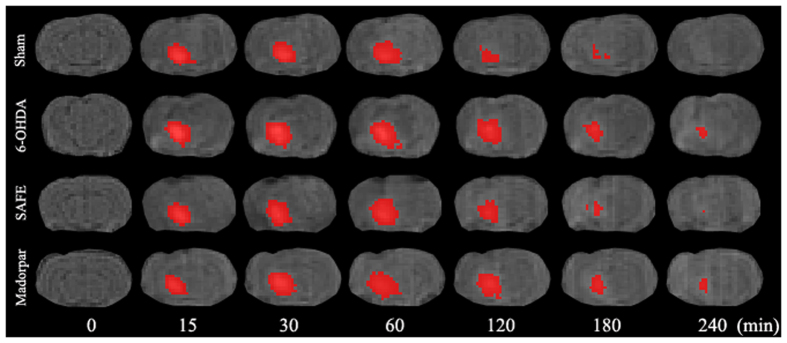
Coronal views of the MRI appearances of Gd-DTPA diffusion in the brain of 6-OHDA-induced rat model of PD after SAFE treatment. The animals were given an intraparenchymal injection of a 2 μl of 10 mM Gd-DTPA in the SN. The anaesthetized rat was placed in a prone position and scanned in the 3.0 Tesla MRI systems machine. For each subject, repeated scans with T1 3D MP-RAGE sequence were performed at 15 and 30 min post-injection and every hour thereafter.

ECS diffusion parameters within the SN are given in Table 1. The free water diffusion coefficient (D) is a constant that was calculated to be 5.18 × 10^−4 ^mm^2^/s in 0.3% agar gel at 37 °C^27^ and the effective diffusion coefficient (D*) was measured in this study. The unilateral damage induced by 6-OHDA in the SN caused a significant decrease in tortuosity (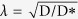; *P* < 0.05, Table 1), and the 70 mg/kg/day SAFE and Madopar treatments significantly restored the decrease of λ (*P* < 0.05 and *P* < 0.01, respectively, Table 1). Figure 7 shows the elimination process of gadolinium-diethylene triamine pentaacetic acid (Gd-DTPA) in the SN. 6-OHDA also markedly reduced the rate constant of clearance (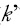) and extended the elimination half-life (t_1/2_) of Gd-DTPA (*P* < 0.05, Table 1). Treatment with 70 mg/kg/day SAFE resulted in trends towards an increase in 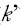 and a shortening of t_1/2_, but these changes were not significant (Table 1).

## Discussion

In this study, two marker compounds (K3R and AYB) and SAFE were evaluated for their neuroprotective effects *in vitro* and *in vivo* using PD models employing rotenone-induced damage to differentiated PC12 cells, and 6-OHDA-lesioning in rats, respectively.

In our previous study, we found that K3R and AYB can bind DJ-1, which is a causative gene product in a familial form of PD and regulates gene expression related to antioxidative stress action^14^. Our previous study also showed that K3R can protect cells from H_2_O_2_-induced injury in a DJ-1-dependent manner, while AYB still exerts protective effects even in DJ-1 knockdown cells^14^. In the present study, a rotenone-induced PD cell model was employed to further assess the protective effects of these two compounds. First, the 3-(4,5-dimethyl-2-thiazolyl)-2,5-diphenyl-2-H-tetrazolium bromide (MTT) assays confirmed that pretreatment with K3R and AYB markedly increased cell viability following rotenone-induced neurotoxicity. Second, using HCA, we demonstrated that neuronal cells pretreated with drug can be exposed to potential toxicants, fluorescently stained for neuronal-specific markers, and quantifiably imaged using image analysis algorithms^28^,^29^. Cell counts, the fluorescence intensity of tubulin and cell area are important indicators that are related to neurotoxicity or neuroprotective effects^28^,^29^. With HCA, we successfully classified the different cells, but K3R and AYB had no effect on the percentage of positive cells. Notably, the increase in fluorescence intensity is probably because cell area greatly decreases as the cells shrink, resulting in an increase in the average fluorescence intensity, thus limiting the value of the parameter in distinguishing between real changes in β-tubulin III fluorescence and apparent changes due to changes in cell area^28^. That is, the intensity of only the positive cells is a more convincing indicator. Recent studies reported that rotenone-induced cytotoxicity is partly caused by increases in the amount of free tubulin and reductions in the amount of polymerized tubulin, which indicates that rotenone destabilizes microtubules^30^. It has been reported that DJ-1 deficiency could cause reduced expression of β-tubulin III in SH-SY5Y and mouse models^31^. Pretreatment with K3R protected the cells (at least the positive cells) against microtubule depolymerization, which might be attributable to the ability of K3R to stabilize DJ-1 by binding to it.Although AYB could also bind DJ-1, it had no effect on the polymerization of β-tubulin. This might be attributed to the fact that the binding between DJ-1 and AYB was nonspecific, and AYB could prevent cells from H_2_O_2_-induced injury in a non DJ-1-dependent manner. Our data showed that pretreatment with AYB increased the cell area, which suggested that AYB could attenuate the shrinkage of cell bodies induced by rotenone. Additionally, using MTT assays, we confirmed that the SAFE prevented PC12 cells from rotenone-induced neurotoxicity at concentrations of 125, 250, and 500 μg/ml (all *P* < 0.01, Supplementary Fig. S2). In summary, our data indicated that K3R, AYB and SAFE prevented rotenone-induced cytotoxicity, and K3R and AYB suppressed the cellular microtubule destabilization and the cell body shrinkage induced by rotenone, respectively.

In general, a standardized herbal extract contains one or more constituents at a particular standard level and provides a batch-consistent product backed by chemical analysis to confirm the presence and the ratio quantity of characteristic plant constituents from one batch to the next^32^. To ensure the quality of SAFE, we set the preliminary quality standard that it consists of more than 60% flavonoids and 5% K3R and AYB. For many extracts, it appears that a number of different constituents work in synergy to produce therapeutic effects^32^. In SAFE, other compounds, such as K3G^15^, K3S^33^, rutin^34^,^35^, Q3G^35^, isorhamnetin^36^ and spermidines^37^ also have many pharmacological effects, including anti-inflammation, anti-oxidant stress, etc. Earlier studies have shown that flavonoids, particularly those of the glycoside flavonoid subclasses found in SAFE, including K3R, K3S, 6HK3G, rutin and Q3G, can more easily enter systemic circulation following oral absorption compared with other flavonoids^38^. Moreover, it has been demonstrated that flavonoids are able to penetrate the blood brain barrier (BBB)^39^. To further increase the absorption and improve the bioavailability of SAFE, a dripping pill form was used in the present study. Dripping pills are rapidly developing dosage forms of TCM that are prepared by blending an herbal extract and a matrix under thermal conditions and then dripping the mixture into a cooling liquid. These processes cause the herbal extract and then the matrix molecules to turn into very fine, tiny crystals that are easily absorbed and thus provide greater bioavailability and stability^40^. Our results demonstrated that the RSD of the pill weight was below 15%, and the disintegration time of pills was below 30 min, criteria which met the CP standards^26^. In short, the quality standard of SAFE was established and the SAFE dripping pills could be used as a dosage form in further animal studies.

Based on our results from the *in vitro* experiments with the marker compounds and SAFE, we chose the 6-OHDA-induced rat model of PD to evaluate the neuroprotective pharmacological effects of SAFE in dripping pill form. Our results revealed that the apomorphine injections caused turning in the 6-OHDA-induced rat model of PD, and 70 mg/kg/day SAFE treatment improved the behavioural performances of the rats. To further confirm the anti-PD effects of SAFE, we also performed immunohistochemistry for TH, which is a marker for dopaminergic neurons and often used to observe the survival of neurons^41^. Our results showed that SAFE may partially restore dopaminergic neuron numbers. α-syn is the major component of Lewy bodies (LBs), which are a central feature of brains with PD and other synucleinopathies^7^,^8^. This protein is distributed on both the exterior and interior of cells, and neither intracellular nor extracellular α-syn overexpression and aggregation exert much influence on the pathogenesis of PD^42^. Recent studies also indicated that some neurotoxins, including MPTP and 6-OHDA, induce α-syn overexpression or aggregation both *in vivo* and *in vitro*^7^,^8^,^43^,^44^. In our study, SAFE administration reduced the overexpression or aggregation of α-syn in the 6-OHDA-lesioned SN. In summary, our results showed that SAFE might protect dopaminergic neurons from 6-OHDA-induced neurotoxicity.

In the present study, we found that 6-OHDA treatment increased the numbers of reactive astrocytes, which agrees with previous findings^4^,^5^,^6^. Astrogliosis is a key player in neuroinflammatory responses and has long been a pathological observation in PD^4^,^5^,^6^. However, astrogliosis is the subject of much debate. On one hand, the activated astrocytes might produce neurotrophic factors and stimulate microglial cells, resulting in the induction of dopaminergic neurons^4^. On the other hand, sustained inflammatory astrogliosis might result in the secretion of a broad array of neurotoxic molecules, including proinflammatory cytokines, chemokines, prostaglandins and large amounts of reactive oxygen and nitrogen species that contribute to the progression of disease states. Based on the above hypothesis, the suppression of astrocyte reactivity has been a therapeutic target for PD^4^,^5^,^6^. The decrease in immunostaining for GFAP following treatment with SAFE suggests that SAFE reversed the astrocyte activation induced by 6-OHDA in the SN. Additionally, Nissl staining revealed that 6-OHDA caused neuronophagia (solid arrows, Supplementary Fig. S3) following neuronal degeneration and neuronal loss. Neuronophagia is characterized by necrotic neurons that are surrounded or obscured by inflammatory cells^45^,^46^. The administration of SAFE decreased the appearance of neuronophagia, which suggests that SAFE might partly reduce inflammation and enhance cell survival in the 6-OHDA-lesioned SN. To some extent, the level of neuronophagia was also attenuated after Madopar treatment, which was related to the suppression of 6-OHDA-induced dopaminergic neuron loss and reactive astrogliosis. Thus, our data indicated that SAFE possesses anti-inflammatory activity, which might be associated with the anti-inflammatory effects of the flavonoids^15^,^16^,^33^.

Diffusion parameters in the ECS of the brain have been shown to exhibit some significant differences in certain brain diseases, such as ischaemia^20^, Alzheimer’s disease (AD)^20^, and PD^47^. Various methods of qualitatively and quantitatively assessing ECS diffusion parameters have been explored, including real-time iontophoresis (RTI), integrative optical imaging (IOI) and MRI^27^. In the present study, we employed the MRI tracer-based method to demonstrate possible changes in extracellular diffusion following treatment with SAFE in the 6-OHDA-induced rat model of PD. This method was reported for the first time by our colleagues^27^,^48^,^49^ and produces diffusion parameter results that are similar to those produced by other methods^27^. Using Gd-DTPA as the probe, this MRI tracer-based method not only provides effective measurements of ECS diffusion parameters but also allows visualization of the dynamic process of brain interstitial fluid drainage^27^. Many pathological processes in the brain are accompanied by astrogliosis and changes in the extracellular matrix, both of which might affect ECS diffusion parameters^20^. First, astrogliosis is associated with an increase in ECS diffusion barriers, as indicated by the increase in λ and the decrease in 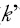
20,22,24,50,51. This occurs because astrogliosis is characterized by astrocytic hypertrophy and increases in the thickness, length, and presumably also number of glial processes that can form the diffusion barriers that are interposed between the cells^50^. Second, the enhanced ECS viscosity arising from the extracellular matrix leads to an increase in λ and a decrease in 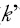
50,51. Although 6-OHDA caused reactive astrogliosis due to the induction of additional diffusion barriers, a decrease in λ was observed in our study, and this decrease is identical to the observations of a previous study of the striatum^47^. A possible explanation is that dopamine neurons are approximately five times longer than astrocytes; thus, the progressive loss of more than 75% of the neurons in the SN was the primary contributor to the reduction in λ^47^. This reduction was reversed following treatment with SAFE or Madopar due to the restoration of the number of neurons. The λ in the 6-OHDA-induced rats treated with Madopar was slightly higher than that in the sham group, which is partially attributable to the fact that glutamate release induced by L-DOPA can also cause an increase in λ^20^,^52^,^53^. Furthermore, we found that 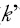 was clearly decreased in the 6-OHDA-lesioned SN, due to the increase in astrogliosis and the potentially elevated levels of extracellular α-syn. A tendency towards an increase in 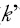 was observed following treatment with SAFE, although SAFE could markedly decrease reactive astrogliosis and overexpression or aggregation of α-syn. An alternative explanation could be that this suppression was not sufficient to increase 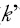. The t_1/2_ was first introduced in our study, and might reflect the drainage speed of the interstitial fluid within the ECS of the brain. The calculation of t_1/2_ can be achieved by quantitatively measuring the extension process of the tracer Gd-DTPA in the SN^27^. Astrocytes might significantly influence the drainage speed, and under neuropathological conditions, reactive astrocytes might contribute to the derangement of interstitial bulk flow and the consequent failure in the clearance of neurotoxic solutes such as Aβ^54^. Our data indicated that 6-OHDA induced an increase in the time required for the elimination of Gd-DTPA from the lesioned SN. The trend towards a reduction in t_1/2_ following SAFE treatment was partially related to the decrease in astrogliosis, which also resulted in an increased fluid clearance of extracellular α-syn. Using the MRI tracer-based method, we first found that 6-OHDA could induce changes in ECS diffusion parameters, including a decrease in λ and 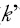 and an increase in t_1/2_ in Gd-DTPA in the 6-OHDA-lesioned SN. The determination of ECS diffusion parameters might provide some information about neuronal loss and astrocyte activation and might also help, to some extent, to prove that SAFE exerted a neuroprotective role in the 6-OHDA-induced rat model of PD.

In summary, SAFE, the effective extract from safflower, was isolated and purified with macropore resin, and the quality standard was preliminarily established. Two marker compounds, K3R and AYB, were proven to prevent rotenone-induced neurotoxicity and suppress the microtubule destabilization and the decrease in cell area induced by rotenone. We further confirmed that SAFE exhibited neuroprotective activities in a 6-OHDA-induced rat model of PD and that these activities were partially mediated by the attenuation of reactive astrogliosis and the suppression of overexpression or aggregation of α-syn. Moreover, to some degree, we demonstrated that the novel MRI tracer-based method utilized in this study could benefit anti-PD drug development in the future via the measurement of ECS diffusion parameters. Additionally, the present study is different and novel compared with our previous work. The extraction process of SAFE was scaled up from 0.5 kg–8 kg for the development of pilot magnification. Two marker compounds, K3R and AYB, were identified and proven to be the important active ingredients in the SAFE. The quality standard was preliminarily established, including the standardized HPLC chromatogram and determination of the marker compounds. Dripping pills of SAFE were also prepared and employed in animal experiments for the first time. The results using a rat PD model induced by 6-OHDA further confirmed the efficacy of SAFE in the treatment of PD. In conclusion, the present study systematically evaluated the extraction process, quality standard, drug form, and *in vitro* and *in vivo* pharmacodynamics of SAFE according to the TCM principles and methods of new drug development.

## Methods

### Materials and Animals

K3R (99% purity) was obtained from the ZeLang group (Nanjing, China), and AYB (90% purity) was supplied by Professor Min Ye. The 6-OHDA was purchased from Sigma (Sigma-Aldrich, St. Louis, MO, USA). Apomorphine was provided by the National Institute for the Control of Pharmaceutical and Biological Products of China.

One hundred approximately 8-week-old male Sprague-Dawley rats weighing 250–300 g were purchased from Charles River Laboratories, Inc. (Beijing, China) with the confirmation number SCXK (Jing) 2012–0001. The rats were maintained at standard room temperature (22 ± 2 °C) and relative humidity (60% ± 10%) on a 12 h light/dark cycle. The rats were allowed free access to food and water throughout the acclimatization and experimental period. All experiments were in strict accordance with the principles and guidelines of the National Institutes of Health Guide for the Care and Use of Laboratory Animals and were approved by the Experimental Laboratory Animal Committee of Peking University.

### Cell culture

Differentiated PC12 cells were purchased from the cell bank of the Chinese Academy of Sciences. The cells were maintained in RPMI 1640 cell culture medium containing 10% foetal bovine serum (Life Tech, Grand Island, NY, USA), 100 U/ml penicillin and 100 mg/ml streptomycin in a water-saturated atmosphere of 5% CO_2_ at 37 °C. Differentiated PC12 cells were sub-cultured every two days.

### Cell viability assay

Differentiated PC12 cells in the logarithmic growth phase were digested with 0.25% trypsin and then resuspended in fresh medium and incubated in 96-well plates. Each well was seeded with 1 × 10^4^ cells in 100 μl. K3R and AYB were dissolved in phosphate buffered saline (PBS). The day after plating, differentiated PC12 cells were pretreated with various concentrations of K3R, AYB or a positive control drug [edaravone (EDA)] for 6 h and treated with 0.5 μm rotenone for 24 h. The control group was exposed to the same solvent. Cell viability was measured with an MTT assay^29^. The absorbance of the control cells was assumed to be 100%.

### Immunofluorescence staining for β-tubulin III and HCA

Differentiated PC12 cells were incubated in 96-well plates coated with 0.1 mg/ml poly-L-lysine, and the treatment compounds were added as well as cell viability assay. Immunofluorescent staining for β-tubulin III (rabbit mAb #2128S, 1:1000, CST) was performed according to a previously described method with slight modifications^29^. The plates were imaged with a PE Operetta High Content System (PerkinElmer, USA). The exposure time was held constant in every assay. A total of 30 fields per well were imaged under 40-fold magnification using two separate filters to capture the nuclei (blue) and β-tubulin III (green). The cells with neurites greater than 38 μm in length were defined as positive cells. The round and pycnotic cells and positive cells were automatically classified with Columbus™ software. The intensity of β-tubulin III in the positive cells and the cell area were also measured. The intensity is actually the signal intensity in arbitrary units and is related to optical density, which depends on the exposure time in the assay.

### Preparation of the SAFE dripping pills

The safflower used in this study was purchased from Sanhe Pharmacy Industry Ltd. (Beijing) and identified by Professor Min Ye. The dried flower petals were twice extracted with 50% EtOH at a ratio of 1:8 (i.e., 1 kg plant material to 8 L 50% EtOH). The collective extract was concentrated in a rotary evaporator and chromatographed on an AB-8 macropore resin column using gradient elution with 15%, 30%, 50%, 70%, and 95% EtOH. The 30% EtOH fraction was collected, concentrated and freeze-dried. To guarantee the quality of SAFE, HPLC was employed to detect the essences of two marker ingredients, K3R and AYB. Qualitative analysis of the components in SAFE was also conducted with LC/MS. Description of the apparatus and chromatographic conditions for HPLC and LC/MS analyses were are provided in the Supplementary materials. Total flavonoids were measured according to the method in the CP^26^.

Preparation and detection of the SAFE dripping pills were executed according to the method in the CP^26^. The SAFE was homogeneously mixed with matrix (PEG 6000) in a water bath at a temperature of 70 °C. The burette was placed 10 cm above the surface of the refrigerant and the drug was dropped into the refrigerant. The pills were collected, and the refrigerant was removed. The weights and disintegration times of the dripping pills were also measured.

### 6-OHDA rat model and rotational behavior

A flow chart of the experiment is shown in Fig. 3a. The surgeries were conducted as previously described^41^. Briefly, the rats were anaesthetized and placed in a stereotaxic frame with an incisor bar positioned 2.4 mm below the interaural line. All rats, with the exception of those in the sham control group, were injected with 6-OHDA (12 μg of 6-OHDA in 6 μl of saline with 0.02% ascorbate), and the rats in the sham group were injected with same volume of saline with 0.02% ascorbate. The stereotaxic coordinates relative to the bregma were as follows: AP = −4.8 mm, ML = −1.8 mm, and DV = −7.8 mm. The injection rate was 1 μl/min, and the needle was kept in place for an additional 10 min prior to slow retraction. After three weeks, the rats were challenged with apomorphine (0.5 mg/kg, i.p.) and allowed to habituate for 10 min. Five minutes after the injection, the full rotations were counted in a cylindrical container in a dimly-lit and quiet room^17^. The rats that exhibited more than 120 rotations in 30 min were used as valid PD models. Next, the sham control rats (n = 10) and valid PD model rats (n = 40) were randomly divided into five groups (n = 10) as follows: Sham group, 6-OHDA group, Low dose of SAFE group (35 mg/kg/day), High dose of SAFE group (70 mg/kg/day) and Madopar group (50 mg/kg/day, L-dopa: benserazide = 4:1). The SAFE dripping pills were suspended in normal saline at a ratio of 10:9 (i.e., 10 mg of pills to 9 ml saline) and were administered by oral gavage once per day for three weeks. The rats in the Madopar group were given Madopar suspended in PEG 6000 solution, and the rats in the sham and 6-OHDA groups were given the PEG 6000 solution only. After the final administration, the rotational asymmetries of each group were scored for 30 min at the sixth week (Fig. 3b).

### Immunohistochemistry

Rats from each group were perfused through the aorta with saline followed by cold 4% paraformaldehyde under deep anaesthesia. After perfusion, the brain was quickly removed, postfixed with 4% paraformaldehyde, and then embedded in paraffin. A series of 10-μm thick coronal sections were cut through the ventral mesencephalon for immunohistochemistry. Four sections were selected from the sections containing the SN of each brain, and all of the sections from all brains were matched as closely as possible. The slides were dewaxed using xylene, rehydrated in an alcohol gradient, incubated with a 1% citrate buffer in 0.1% Triton X100, and then washed with PBS solution for 8 minutes to unmask the antigens. TH (rabbit pAb #sc-14007, 1:200, Santa Cruz Biotechnology), α-syn (rabbit mAb #4179S, 1:200, CST) and GFAP (mouse mAb #3670S, CST) immunohistochemistry was performed using previously published 3, 3′-diaminobenzidine (DAB) protocols^7^. After sealing the slides, images were obtained using an Olympus light microscope (Olympus IX71, Japan). For each section, the numbers of TH-immunopositive neurons and GFAP-positive astrocytes were manually counted at 40-fold and 100-fold magnification, respectively. TH-positive cells in the SN were evaluated in both lesioned and intact hemispheres, and the data are expressed as a percentage of the corresponding area from the intact side (% of contralateral control). GFAP-positive astrocyte counts were expressed as a percentage of the lesioned SN from the sham control (% of sham). For semi-quantitative determination of α-syn immunoreactivity, the region of interest in the lesioned SN was imaged at 200-fold magnification. α-syn staining was semi-quantified using Image J software 1.43 (NIH) to determine the percentage of area containing-positive α-syn staining in the entire area of the captured image (dashed line, Fig. 5a)^55^. The investigator was unaware of the experimental groups.

### The measurement of ECS diffusion parameters with the MRI tracer-based method

ECS diffusion parameters were determined with the MRI tracer-based method^27^,^48^,^49^. After the final administration, a volume of 2 μl Gd-DTPA solution (10 mM) was microinjected over 5 min, followed by a 5-min waiting period to avoid dorsal reflux along the needle track. The injection site shared identical stereotaxic coordinates with the last 6-OHDA microinjection. The anaesthetized rat was placed in the prone position and scanned with a T1-weighted three-dimensional magnetization prepared-rapid acquisition gradient echo (T1 3D MP-RAGE) sequence in a 3.0 Tesla MRI systems machine (Magnetom Trio, Siemens Medical Solutions, Erlangen, Germany). The parameters for the T1 3D MP-RAGE sequence have been previously described^27^. For each subject, repeated scans were performed at 15 and 30 min post-injection and every hour thereafter.

The calculations of the diffusion parameters have also been described in previous reports^27^,^48^,^49^. A piece of MATLAB-based software was developed^27^,^49^ to co-register the magnetic resonance (MR) images of the same rat before and after the injection. The pre-injection images were subtracted from the post-injection images to identify the areas that were enhanced by Gd-DTPA. According the modified diffusion equation^27^,^48^,^49^ and the standard least squares fitting technique, the MATLAB-based software was employed for the computation of the diffusion parameters including λ, 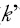 and t_1/2_. Based on the linear relationship between MR signal enhancement (Δ*Signal intensity* (*SI*) = *SI*_Pre-injection_−*SI*_post-injection_) and Gd-DTPA concentration (C) as assessed with T1 3D MP-RAGE^48^, the total amount of Gd-DTPA was calculated for each time point. The clearance of Gd-DTPA from the brain obeys a first order kinetic equation; thus, the t_1/2_ could be calculated.

### Statistical analyses

The data are represented as the mean ± SEM. The significances of the differences between groups were evaluated by one-way ANOVA using the Student-Newman-Keuls post hoc test. Differences were considered significant when *P* < 0.05.

## Additional Information

**How to cite this article**: Ren, R. *et al.* Neuroprotective Effects of A Standardized Flavonoid Extract of Safflower Against Neurotoxin-Induced Cellular and Animal Models of Parkinson,s Disease. *Sci. Rep.* **6**, 22135; doi: 10.1038/srep22135 (2016).

## Supplementary Material

Supplementary Information

## Figures and Tables

**Table 1:**
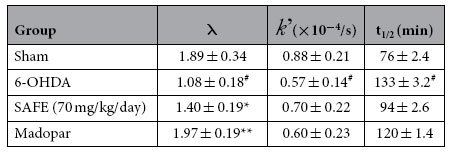
Effects of SAFE on ECS diffusion parameters in the SN of 6-OHDA-induced rat model of PD.

Tortuosity 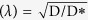; 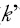 = the rate constant of clearance. Based on the linear relationship between MR signal enhancement and Gd-DTPA concentration (C) as assessed with T1 3D MP-RAGE, the total amount of Gd-DTPA was calculated for each time point. The clearance of Gd-DTPA from the brain obeys a first order kinetic equation; thus, the t_1/2_ could be calculated. These parameters were calculated with a piece of MATLAB-based software. Statistical analyses were performed using one-way ANOVA. Data are the mean ± SEM, n = 3–4, ^#^*P* < 0.05 vs. Sham group; *P < 0.05, **P < 0.01 vs. 6-OHDA group.
