# Supplementary material for: Neuroprotective Effects of A Standardized Flavonoid Extract of Safflower Against Neurotoxin-Induced Cellular and Animal Models of Parkinson’s Disease
Source: Sci Rep. 2016 Feb 24;6:22135. doi: 10.1038/srep22135 (PMC4764910; doi:10.1038/srep22135)
Supplement: Supplementary Information [file srep22135-s1.pdf]

Neuroprotective Effects of a standardized flavonoid extract of safflower against Neurotoxin-Induced Cellular and Animal Models of Parkinson's Disease

Rutong Ren[a, b], Chunyan Shi[c, d], Jing Cao[b], Yi Sun[a, b], Xin Zhao[a, b], Chen Wang[a, b], Yongfei Guo[a, b], Hui Lei[a, b], Hanjie Jiang[a, b], Nuramatjan Ablat[a, b], Jiamin Xu[a, b], Wan Li[a, b], Yingcong Ma[b], Xianrong Qi[b], Min Ye[b], Xiaoping Pu[a, b,*], Hongbin Han[c, d, *]

a Department of Molecular and Cellular Pharmacology, School of Pharmaceutical Sciences, Peking University, Beijing 100191, China

b State Key Laboratory of Natural and Biomimetic Drugs, Peking University, Beijing 100191, China

c Beijing Key Lab of MRI Device and Technique, Beijing 100191, China

d Department of Radiology, Peking University Third Hospital, Beijing 100191, China

*Corresponding author:

Xiaoping Pu, E-mail: pxp123@bjmu.edu.cn

Hongbin Han, E-mail: hanhongbin@bjmu.edu.cn

Table S1 | The important parameters for evaluating ECS

Symbol	Name	Unit	Definition
α	Volume fraction	Dimensionless	Ratio of the ECS occupies of the total brain volume
D*	Effective diffusion coeffcient	mm^2/s	$D^* = D\backslash\lambda^2$, Diffusion rate of a substance in a diffusion-limited medium, such as the brain
λ	Tortuosity	Dimensionless	Pathway of a substance diffusing from one point to another in the brain ECS, the length is equal to or longer than the straight line distance between these two points
k'	Clearance	Mmol/L	Loss or clearance of a substance from the ECS, into cells, across the blood brain barrier (BBB), or by degradation through enzymatic attack or other processes

D is a free diffusion coefficient (mm^2 /s) and defined as diffusion rate of a substance in a free medium, such as the diluted agarose hydrogel.

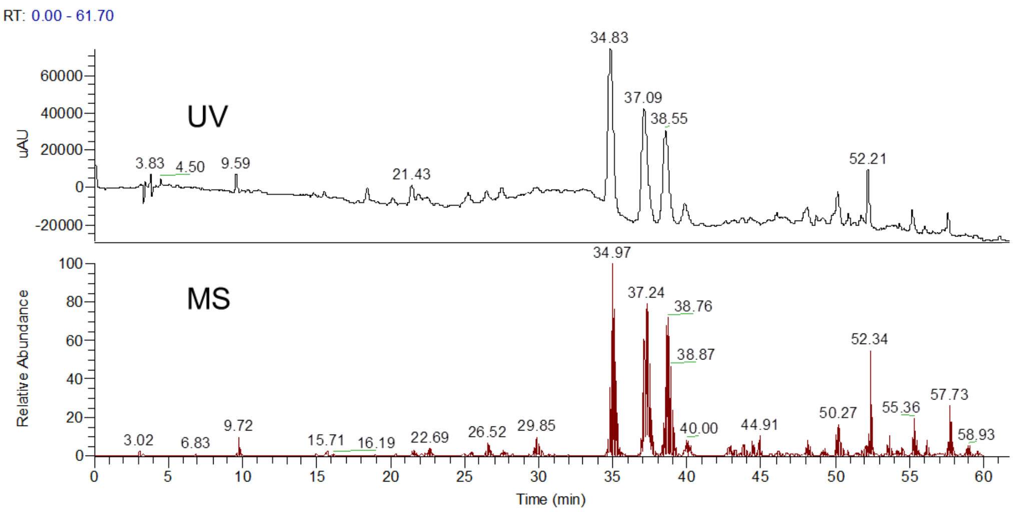

Figure S1 | The chromatogram and mass spectrum of SAFE.

Table S2 | The identified compounds in the SAFE with LC/MS analysis

No.	RT(min)	[M-H]-(m/z)	MSn(m/z)	Identification
1	9.72	611	MS^2[611]:491 ,473,325 MS^3[491]:473,283	hydroxysafflor yellow A (HSYA)
2	14.93	771	MS^2[771]:609,463,301MS^3[609]:301	6-hydroxykaempferol 3-O-rutinoside-7-O-glucoside (6H3R7G) or 6-hydroxykaempferol 3-O-β-D-rutinoside-6-O-β-D-glucoside (6H3R6G)
3	15.71	625	MS^2[625]:463, MS^3[463]:301	6-hydroxykaempferol 3,7-di-O-glucoside (6H3G) or 6-hydroxykaempferol 6,7-di-O-β-D-glucoside (6H6G)
4	20.4	609	MS^2[609]:301	6-hydroxykaempferol 3-O-β-D- rutinoside (6H3R) or quercetin 3-O-rutinoside (rutin)
5	21.55	463 1087	MS^2[463]:301; MS^3[301]:255,271 MS^2[1087]:625,461 MS^3[625]:463,301 MS^4[463]:301	6-hydroxykaempferol 3-O-β-D-glucoside (6HK3G)
6	22.08	609	MS^2[609]:429,284,339,447 MS^3[284]:338	kaempferol 3-O-β-D-sophoroside (K3S)
7	22.69	1043	MS^2[1043]:1025,923,1007,449 MS^3[1025]:1007,863,671 MS^2[923]:449,527,759	isomer of anhydrosafflor yellow B (AYB)
8	21.34	609	MS^2[609]:301,489,549,591 MS^3[301]:273,255,179	quercetin 3-O-rutinoside (rutin)
9	26.52	1043	MS^2[1043]:1025,923,1007,449 MS^3[1025]:1007,863,671 MS^2[923]:449,527,759	isomer of anhydrosafflor yellow B (AYB)
10	27.59	463	MS^2[463]:301,180,393 MS^3[301]: 179,254,271	qucertin-3-O-glucoside (Q3G)

11	29.69	449	MS^2[449]:287,255,167 MS^3[287]:181,259,241	4',5-dihydroxyl-7-O-glucopyranosyl flavanone (4D7GF)
12	34.97	593	MS^2[593]:285 MS^3[285]:257,229,213,197	kaempferol 3-O-rutinoside (K3R)
13	37.24	623	MS^2[623]:315, 300 MS^3[315]:300	6-hydroxyapigenin 6-O-β-D-glucoside-7-O-β-D-glucuronide (6H6G7G)
14	38.76	1043	MS^2[1043]:1025,923,449 MS^3[1025]:1007,863 MS^4[863]:845,671,771,287	anhydrosafflor yellow B (AYB)
15	40	447	MS^2[447]:315, 300 MS^3[315]:300	kaempferol 3-O-glucoside (K3G)
16	43.78	449	MS^2[449]:287, 253 MS^3[287]:181,167	4',5-dihydroxyl-6-O-glucopyranosyl flavanone (4D6GF) or its isomer
17	44.43	449	MS^2[449]:287, 253 MS^3[287]:181,167	4',5-dihydroxyl-6-O-glucopyranosyl flavanone (4D6GF) or its isomer
18	44.91	836	MS^2[836]:790 MS^3[790]:772,564,451	isorhamnetin
19	51.47	491	MS^2[491]:287 ,181,431 MS^3[287]:181,259	4',5-dihydroxyl-7-O-[6''-acetyl(1→6)]-glucopyranosyl flavanone (4D76GF)

Also, in the SAFE there are still some unknown compounds that still need identification

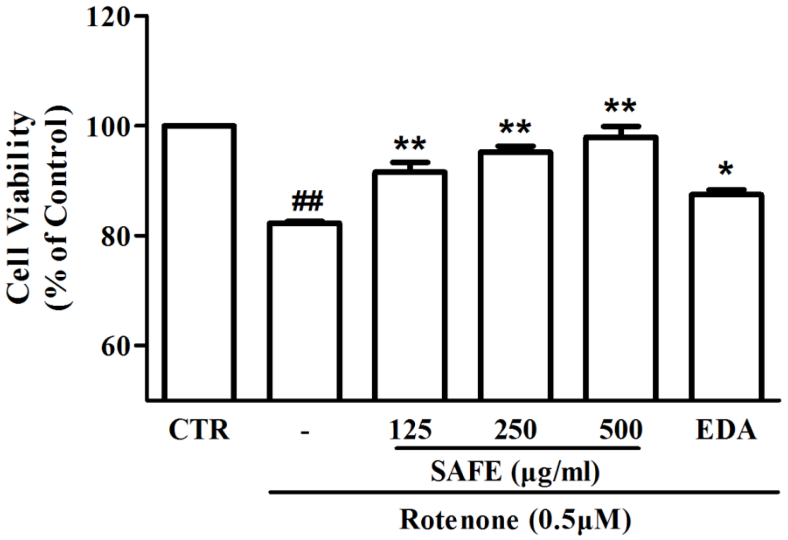

Figure S2丨Effects of SAFE on cell viability of rotenone-induced differentiated PC12 cell damage. Cells were pretreated with SAFE (125, 250 and 500 μg/ml) for 6 h before rotenone treatment for 24 h. Data are the mean ± SEM, n = 3, ##*P* < 0.01 vs. untreated control (CTR) group; **P* <0.05, ***P*<0.01 vs. rotenone group.

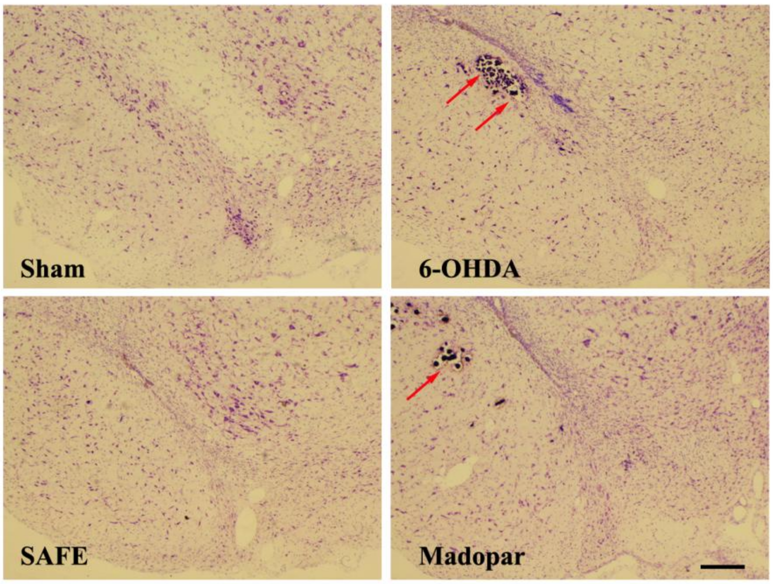

Figure S3丨Multiple foci of neuronophagia (solid arrows) as denoted by necrotic neurons surrounded or obscured by inflammatory cells. Bar = 200 μm. The sections of SN were prepared and picked up as described as immunohistochemistry and then the sections was stained with 0.5% cresyl violet acetate in the SN.

Apparatus and chromatographic conditions for HPLC and LC/MS. An Agilent 1100 HPLC system equipped with a quaternary solvent delivery system, an autosampler and a DAD detector was used. Separation was achieved on an Alltima-C18 column (5 μm, 250 mm × 4.6 mm, Agilent). The mobile phase consisted of A (0.3% aqueous methanoic acid) and B (Acetonitrile: methanol = 9:1), which were applied in the gradient elution as follows: 0-5 min：10-14% B；5-25 min：14-17% B；25-35 min：17-18% B；35-45 min：18-25% B；45-55 min：25-30% B; 55-60 min: 30 % B. Each run was followed by equilibration time of 5 min. The flow rate was 1.0 ml/min. The column temperature was set at 30◦C. Ultraviolet (UV) spectra were monitored at 345 nm. The injection volume was 10 μl. The data were collected and analyzed with Chemstation 10.02 software.

For MS analysis, the HPLC conditions were the same as described and the mass spectrometer was operated in (−)-ESI mode. Ultra-high purity helium and high-purity nitrogen was used as the collision gas and the nebulizing gas, respectively. The optimized parameters were as follows: ion spray voltage, 3.5 KV; sheath gas, 50 arbitrary units; auxiliary gas, 10 arbitrary units; capillary temperature, 350 ℃; capillary voltage, −49.0 KV; tube lens offset voltage, −36.0 V. Mass spectra were recorded in the range of m/z 130-1000. The collision-induced dissociation energy was adjusted to 40%, and the isolation width of precursor ions was 2.0 mass units. Data were processed by Xcalibur 1.4 software (ThermoFisher).
